# Advancing health equity in proactive health management: from data underrepresentation and algorithmic bias to a closed-loop governance framework

**DOI:** 10.3389/fpubh.2026.1863050

**Published:** 2026-06-17

**Authors:** Qiming Zhao, Wenhui Jiang, Chen Zhang, Minglian Ouyang, Nan Wu, You Guo

**Affiliations:** 1 Medical Big Data and Bioinformatics Research Center, First Affiliated Hospital of Gannan Medical University, Ganzhou, China; 2 School of Public Health and Health Management, Gannan Medical University, Ganzhou, China; 3 First School of Clinical Medicine, Gannan Medical University, Ganzhou, China; 4 Ganzhou Key Laboratory of Medical Big Data, Ganzhou, China; 5 Gannan Subcenter for Medical Genomics, National Genomics Data Center, Ganzhou, China

**Keywords:** algorithmic bias, artificial intelligence, health equity, patient-generated health data, proactive health management, social determinants of health

## Abstract

Proactive health management (PHM) relies on wearable devices, mobile health applications, and artificial intelligence (AI) to continuously monitor the health status of individuals, thus supporting risk stratification and early intervention. The effectiveness of such techniques fundamentally depends on whether training data adequately represents the target population. Structural data gaps in patient-generated health data (PGHD), driven by social determinants of health (SDoH) and the digital divide, result in the systematic underrepresentation of high-risk groups in training data. This underrepresentation cascades through model development and deployment, leading to prediction bias, risk omission in vulnerable populations, and misallocation of health resources. To synthesize current evidence on this topic, we conducted a narrative review searching PubMed, Web of Science, and Scopus for peer-reviewed literature published between January 2015 and February 2026, using terms related to proactive health management, artificial intelligence, algorithmic bias, and health equity. A total of 108 studies meeting predefined inclusion criteria were included and synthesized thematically. This review analyzes the mechanisms driving insufficient data representativeness in PHM and traces how prediction bias accumulates across model development and deployment, resulting in health inequities. We identify that equity risk in PHM stems from the interplay of three factors: social inequality structures, data collection mechanisms, and predictive target design. Because these drivers operate across the entire PHM lifecycle, addressing equity requires integrated, system-wide governance rather than isolated technical adjustments. This paper therefore proposes a comprehensive closed-loop governance framework encompassing targeted data collection, fairness constraint mechanisms, pre-deployment subgroup audits, and post-deployment continuous monitoring. By transforming fairness from a technical condition into a core systemic principle, this framework aims to ensure health equity throughout the PHM lifecycle.

## 1 Introduction: the equity of proactive health management

Hospital-centered passive diagnosis and treatment is gradually giving way to proactive health management (PHM)—a continuous, data-driven paradigm that uses wearable sensors, mobile health applications, and artificial intelligence (AI)-based prediction models to support risk stratification, early detection, and timely intervention before disease onset (1–3). Traditional clinical care relies primarily on intermittent cross-sectional records generated during healthcare encounters. In contrast, PHM supports clinical decision-making with longitudinal and continuous multimodal data, covering real-time physiological signals collected by wearable devices, behavior patterns recorded by mobile platforms, and out-of-hospital environmental exposure information (4, 5). These data help capture subtle physiological changes in the subclinical stage, create conditions for early intervention, and promote the shift of health services from treating diagnosed diseases to preventing disease onset (6).

The generalization ability of prediction models is constrained by the representativeness of training data, which has been extensively documented in previous studies (7). Patient-generated health data (PGHD)—health-related information generated by individuals outside formal clinical encounters, including wearable sensor signals, self-reported symptoms, behavioral activity patterns, and contextual information—is a central data source for PHM, yet non-random missingness is common in real-world applications (8). These omissions are not randomly generated technical noise, but are systematically structured by social determinants of health (SDoH; the non-medical social, economic, and environmental conditions that shape health outcomes) and the digital divide—unequal access to digital devices, internet connectivity, and digital literacy—both of which determine who can generate and benefit from digital health data (9–11). Evidence from recent digital health equity studies indicates that access to high-quality wearable devices, stable internet connections, and sustained engagement with digital health tools is socially patterned rather than evenly distributed. Younger individuals, urban residents, and people with higher socioeconomic status are generally more likely to adopt and continuously use digital health technologies, whereas older adults, rural residents, low-income groups, and individuals with limited digital literacy face persistent barriers to participation (12–15). Although the magnitude and form of these disparities vary across health systems and countries, their consequence for PHM is consistent: structurally disadvantaged groups are less likely to be represented in continuous PGHD streams, thereby increasing the risk of biased model training and inequitable prediction performance (16).

The fundamental challenge confronting PHM is therefore not the absolute volume of available data, but whether the data collected adequately represent the populations at greatest clinical need. When certain subgroups are insufficiently represented on key variables, models may fail to capture their distinct risk patterns, manifesting as elevated false-negative rates, compromised predictive calibration, or unstable threshold performance within these populations (17). For PHM systems reliant on automated early warning, the implications of these disparities extend far beyond abstract statistical metrics. They determine who will be identified by the system, who can receive continuous follow-up, and who has access to preventive resources. When resource allocation is directly linked to model output, the structural inequalities embedded in data collection and service access pathways are further amplified following algorithm deployment (18).

The importance of algorithmic fairness and health equity in AI applications in healthcare has attracted attention, but most of the existing research focuses on bias correction in the model training or output stage (19). From data representation gaps to algorithmic bias, resource mismatches, and erosion of institutional trust, this complete chain lacks systematic examination. Digital health tools are deeply embedded in daily life, and there exists a reinforcing feedback loop between data generation and service provision. Addressing this gap requires a governance framework that integrates human-in-the-loop (HITL) methods, continuous audit mechanisms, and intervenable nodes into system design from the outset, rather than applying corrective measures retrospectively after deployment.

This review addresses three interrelated questions: how structural and technical deficits produce non-random underrepresentation in PGHD and related health data; how upstream data gaps propagate and amplify into performance disparities and inequitable outcomes across model development and deployment; and what corrective strategies are available at the data, algorithm, and governance levels, and under what real-world conditions these strategies can be effectively implemented. The review adopts a global rather than country-specific analytical scope, synthesizing evidence across diverse health system contexts, while acknowledging that PHM research and governance discourse have been most extensively developed in high-income settings; where available, evidence from low- and middle-income contexts is incorporated. This review aims to provide a coherent analytical framework for understanding the health equity challenges in PHM and to inform equitable governance practices in the field of digital health.

## 2 Methods

This review employs a narrative synthesis approach to examine evidence on data representativeness deficits, algorithmic bias mechanisms, and health equity implications in PHM. A narrative rather than systematic synthesis was adopted given the considerable heterogeneity in study design, application domain, and outcome definition across the included literature, which precludes quantitative meta-analysis. The review process was guided by the Scale for the Assessment of Narrative Review Articles (SANRA) criteria to enhance methodological transparency (20).

A systematic literature search was conducted across three electronic databases—PubMed, Web of Science, and Scopus—covering publications from January 1, 2015 to February 28, 2026. Searches were structured around three thematic domains: PHM and wearable health technologies (Domain 1), AI and machine learning (ML) (Domain 2), and algorithmic bias and health equity (Domain 3), combined using the Boolean operator AND. Domain 1 included the following terms: “proactive health management” OR “wearable device*” OR “patient-generated health data” OR “PGHD” OR “mobile health” OR “mHealth” OR “remote patient monitoring”. Domain 2 included: “artificial intelligence” OR “machine learning” OR “deep learning” OR “predictive model*” OR “algorithmic” OR “clinical decision support”. Domain 3 included: “algorithmic bias” OR “algorithmic fairness” OR “health equity” OR “health disparit*” OR “digital divide” OR “social determinants of health” OR “underrepresentation.” In PubMed, searches were supplemented with the following MeSH terms: Health Equity [MeSH], Artificial Intelligence [MeSH], Wearable Electronic Devices [MeSH], Healthcare Disparities [MeSH], and Social Determinants of Health [MeSH].

Studies were included if they: (a) addressed AI or ML predictive model applications in PHM or related preventive health contexts; (b) explicitly discussed data underrepresentation, algorithmic bias, fairness evaluation, or health equity, and provided substantive conceptual or empirical evidence relevant to the thematic narrative; and (c) were peer-reviewed original articles, systematic reviews, scoping reviews, or narrative reviews published in English. Studies were excluded if they: (a) were editorials, conference abstracts, letters, or non-peer-reviewed publications; (b) focused solely on AI or ML model performance without any discussion of data representativeness, algorithmic bias, or health equity; (c) were published in languages other than English; or (d) were published outside the specified date range.

Following the database search, title and abstract screening was performed independently by two reviewers (QZ and WJ), followed by a full-text assessment of all potentially eligible records to evaluate both methodological fit and thematic contribution to the closed-loop governance framework. Disagreements were resolved through structured discussion; where consensus could not be reached, a third reviewer (YG) served as arbiter. In addition to database searches, relevant studies were identified through manual screening of reference lists and backward and forward citation tracking of key publications, including foundational works published prior to the primary search window, to capture seminal and highly relevant studies not retrieved by the initial search strategy; these were integrated based on their thematic and methodological relevance to the narrative synthesis. A total of 108 studies were included in the final narrative synthesis. To further enhance methodological transparency and reproducibility, the platform-specific formatted search strings applied in each database are provided in Supplementary material S1.

Evidence was synthesized thematically across three pre-specified analytical domains: (1) mechanisms of bias formation in PHM data and models; (2) inequitable health consequences of algorithmic bias; and (3) governance and mitigation strategies at the data, algorithm, and system levels. This problem-oriented narrative structure enabled coherent integration of evidence across diverse study designs and health system contexts.

## 3 The formation mechanism of predictive deviation in proactive health management

The risk of algorithmic bias and resulting health inequities in PHM does not stem from isolated technical errors. Differences gradually accumulate in the process of data collection, model training and clinical deployment (21–23). Reconceptualizing predictive bias as a cumulative lifecycle trajectory, our framework demonstrates how upstream representativeness deficits at the data collection stage are not merely carried forward, but are actively amplified during model development and ultimately institutionalized through clinical deployment decisions. By structurally linking data infrastructure inequalities, empirical risk minimization (ERM), and misaligned prediction targets, this perspective captures the complete operational mechanism through which socio-structural disadvantages are translated into disparate preventive care.

### 3.1 Representativeness deficits: non-random missingness in PGHD

PHM relies on the physiological and behavioral data continuously generated by patients. The absence of PGHD data flow is rarely random, which is directly related to SDoH and the digital divide (24–27). There are differences in equipment ownership and basic digital literacy among different groups of people, and the continuous use of mobile health applications also varies according to income level, geographical location and educational background. The problem is not only that the sample size is small, but also that the target population is not fully covered: the patients who need clinical intervention the most are often the most likely to be absent from the training cohort.

The data gap is not only reflected in the acquisition level, but also the measurement quality itself varies among different groups of people. Photoplethysmography (PPG)-based heart rate estimation may yield reduced accuracy in individuals with darker skin tones due to melanin interference with optical signal acquisition (28, 29). Long-term monitoring is also affected by signal noise, transmission interruption and decreased patient compliance, and the stability of longitudinal risk assessment is weakened (30). Data continuity and technical reliability are as important as initial collection.

A concrete international example is pulse oximetry, which shares optical sensing principles with PPG-based monitoring technologies. Recent systematic reviews have shown that pulse oximeters may overestimate arterial oxygen saturation in individuals with darker skin tones, increasing the risk of unrecognized hypoxemia (31, 32). This issue became especially visible during the COVID-19 pandemic, when home-based oxygen monitoring and remote pulse oximetry were widely used. In the United Kingdom, evidence from pulse oximeters used in home monitoring and National Health Service (NHS) clinical settings has raised specific concern that skin-tone-related measurement error may contribute to unequal recognition of respiratory deterioration in ethnically diverse populations (33). This case is directly relevant to PHM because biased physiological measurements can be transferred from clinical monitoring devices into wearable sensors and remote monitoring platforms, embedding inequity at the earliest stage of data generation.

When multiple social disadvantages are superimposed, these gaps are further widened (34). Electronic health records (EHR) add additional complexity to this process: the recordings reflect historical differences in access to medical services and diagnosis paths, rather than objective biological needs (35, 36). In low- and middle-income countries, EHR-derived labels may be particularly vulnerable to bias because chronic diseases such as hypertension, diabetes and chronic kidney disease are often underdiagnosed, undertreated, inconsistently coded, or documented only after advanced disease progression. Recent global evidence shows that diagnosis, treatment and control coverage for major non-communicable diseases remain highly uneven across countries and income groups, particularly for hypertension and diabetes (37, 38). Structural inequalities become embedded in algorithmic systems through training data, and historical bias persists in the model.

### 3.2 Bias amplification: mismatch of distribution in model development

After the upstream data gap enters the model development stage, it does not self-correct but is instead further amplified. The distribution of health data in the real world is rarely stable (39), and models trained on stable behavioral patterns may fail to generalize in environments with limited resources or data fluctuations. Performance declines, sensitivity is reduced, calibration is poor, and risk stratification becomes unreliable (40, 41). These issues are more prominent when cross-regional deployments or algorithms are transferred from tertiary hospitals to primary clinics. The actual significance of the same decision-making threshold is not the same in different clinical environments (42).

Real-world validation studies illustrate that this problem is not merely theoretical. In the United Kingdom, earlier cardiovascular risk prediction tools, including models derived from the Framingham Risk Score tradition, were developed largely from populations that did not fully represent the ethnic diversity of contemporary UK primary care. Subsequent evaluation in multi-ethnic UK cohorts showed variable calibration across White European, South Asian, and Black British populations (43). The iterative development of QRISK-family algorithms, including QRISK3 and QR4, which incorporated variables such as ethnicity, deprivation and comorbidities and explicitly assessed subgroup calibration (44), should not be interpreted as evidence that distribution shift has been eliminated. Rather, it demonstrates that models derived from large but demographically uneven datasets require active subgroup validation and recalibration before they can be safely applied to diverse populations. The need for successive algorithmic updates reflects a persistent challenge for PHM: when patient profiles, comorbidity patterns, documentation practices and data completeness differ from the derivation cohort, the same model may produce systematically different levels of error across groups.

Similar problems have been reported in sepsis prediction. External validation studies of machine-learning-based sepsis prediction models have shown that algorithms developed in one hospital or health system may show degraded discrimination or calibration when transferred to other clinical settings (45). These findings demonstrate how differences in physiological baselines, disease burden, clinical workflows, documentation practices and data infrastructure can produce distribution shift and undermine model transportability.

Majority dominance exacerbates this problem. ERM training optimizes average performance across the full training population, meaning that high-frequency patterns from majority groups receive disproportionate weight while risk signals unique to minority or underrepresented groups may exert limited influence on the learned decision boundary and be reduced to statistical noise (46). Recent studies on fair empirical risk minimization and fairness in healthcare AI further indicate that aggregate optimization alone is insufficient to guarantee reliable subgroup performance when training data are imbalanced or subgroup risks are poorly represented (47, 48). A model may show a high Area Under the Receiver Operating Characteristic Curve (AUROC) in the overall evaluation, but for individuals of long-tailed populations, the same model may be accompanied by a high false-negative rate (FNR) and poor calibration (49). The overall performance indicators mask these fairness deficits and equity risks, and the evaluation at the subgroup level constitutes a necessary condition for the PHM system.

The degree of bias amplification varies according to the specific clinical task. Simple predictive tasks performed in a stable environment have limited subgroup differences; models of continuous behavior monitoring and long-term compliance are more prone to problems. Technical bias is constrained by deployment conditions, not static model defects.

### 3.3 Value misalignment: assumptions in proxy variables and prediction targets

The definition of the prediction target will also introduce bias. Researchers often measure health status by proxy indicators such as medical expenditure or service utilization frequency, and it is difficult to actually measure real health status. Such indicators reflect economic capacity and insurance coverage, not true disease burden (50, 51). Vulnerable groups spend less on medical care and have fewer medical visits, even when facing equivalent or greater health risks. Therefore, the prediction system may systematically underestimate the actual clinical needs of these populations. Obermeyer et al. demonstrated that using medical costs as a risk indicator will systematically underestimate the disease burden of poor patients and redistribute resources to patients with better actual health status (52).

A parallel example outside the United States involves estimated glomerular filtration rate (eGFR) equations. In the United Kingdom, the historical use of ethnicity-based correction factors in kidney function estimation has been questioned because it may alter kidney function estimates for people of Black ethnicities and affect clinical interpretation, referral thresholds and eligibility assessment for specialist care or transplantation (53). This case illustrates how value misalignment can arise from clinical conventions embedded in proxy variable construction, independently of data volume or model complexity. In other words, bias may be introduced not only through missing data or model optimization, but also through the clinical assumptions used to define what the algorithm is asked to predict.

The setting of clinical thresholds also needs attention. Based on the risk threshold of general population data calibration, the effect is not ideal in specific age, gender or socioeconomic groups (54). Different groups of people adopt unified decision-making rules, and the distribution of results will be unequal. The root cause of the bias appears earlier, originating in the stage of label definition and proxy variable selection, which are often regarded as routine methodological conventions and are not sufficiently examined (55).

**Figure 1:**
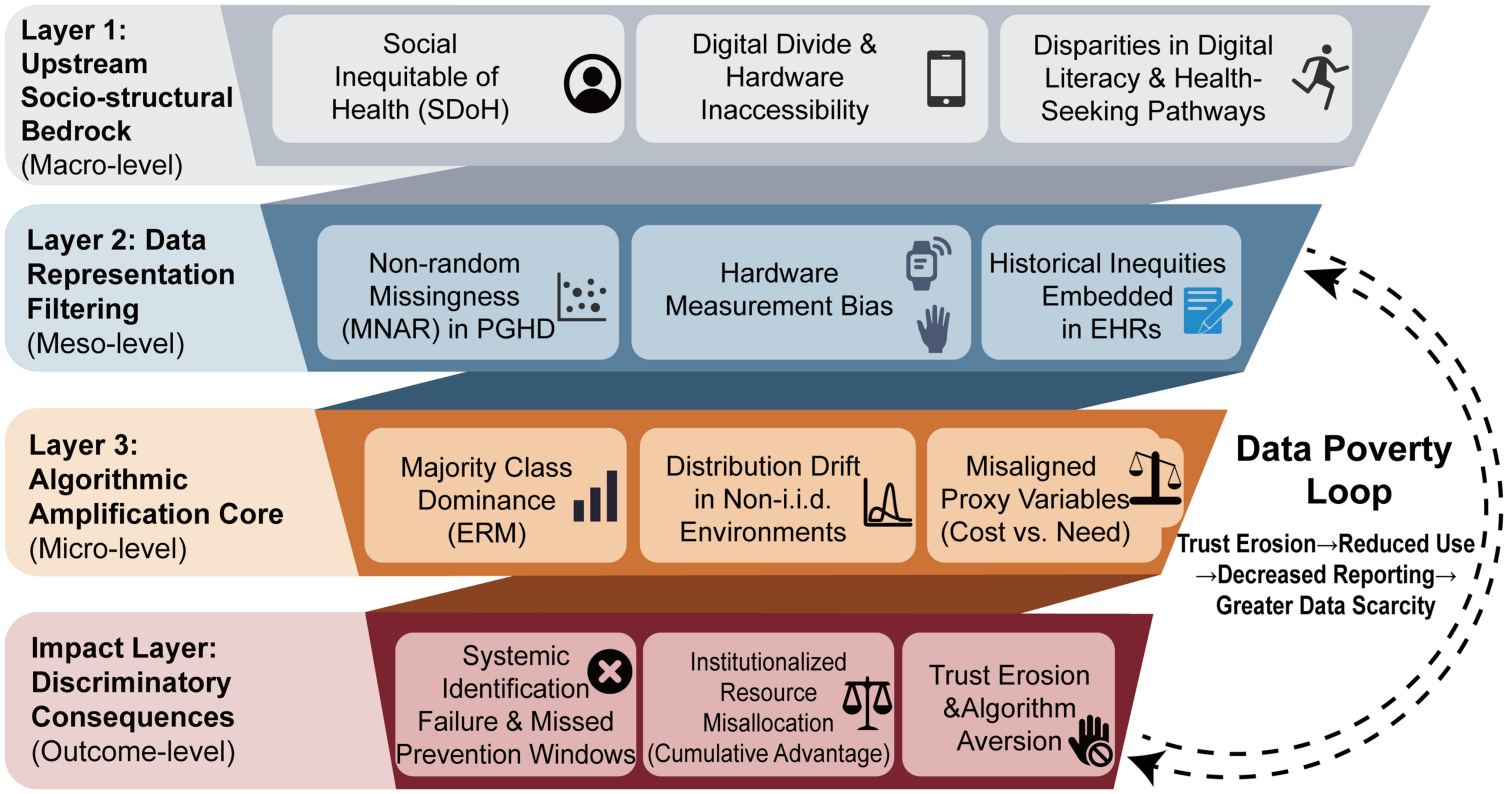
Multilevel pathways from insufficient data representativeness to predictive bias and inequitable consequences in proactive health management. This figure illustrates how data gaps and selection bias accumulate through successive layers to produce discriminatory consequences in proactive health management. Layer 1 (macro level) encompasses upstream structural factors including social determinants of health, the digital divide and hardware accessibility constraints, and disparities in digital literacy and health-seeking behaviors. Layer 2 (meso level) represents how these structural factors translate into data-level biases: non-random missingness in patient-generated health data, systematic measurement error across devices, and persistent health inequities in electronic health records. Layer 3 (micro level) shows how these representativeness gaps are amplified through algorithmic processes. Majority class dominance under empirical risk minimization, distribution shift in non-independent and identically distributed (Non-IID) environments, and the use of proxy variables that correlate with protected characteristics all intensify disparities across population subgroups. The outcome layer depicts downstream consequences: systemic failures in risk detection and missed prevention opportunities for vulnerable populations, institutionalized resource misallocation favoring well-represented groups, and progressive erosion of patient trust and engagement. The feedback loop (dashed arrows) indicates how reduced device usage and reporting adherence among disenfranchised populations further exacerbate data scarcity and perpetuate the cycle of algorithmic bias. PHM, proactive health management; SDoH, social determinants of health; PGHD, patient-generated health data; EHRs, electronic health records; ERM, empirical risk minimization; Non-i.i.d., Non-independent and identically distributed; MNAR, missing not at random.

The above three links —representativeness deficits, bias amplification and value misalignment— reveal how predictive bias extends throughout the entire lifecycle of PHM. Table 1 summarizes the action path of each mechanism and its downstream impact; Figure 1 shows the nature of the superposition of biases and the feedback loop driven by data scarcity.

**Table 1:** Internal mechanisms of predictive bias in proactive health management and their operational pathways.

Mechanism category	Source of bias	Manifestation in PHM	Impact on performance	Key affected groups	PMID
Socio-structural	SDoH and digital divide	Non-random missingness of PGHD; monitoring interruptions	Reduced generalizability; elevated false-negative rates	Low-SES populations; older adults; marginalized populations	35,754,462
Hardware-driven	Sensor Performance	Decreased PPG accuracy in individuals with darker skin tones	Increased signal noise; unstable risk thresholds	Individuals with darker skin tones	41,306,936
Technological	Majority Class Dominance	ERM-driven models ignore minority-specific patterns	High aggregate performance but subgroup failure	Minority or underrepresented subgroups	37,521,051
Value-based	Proxy Variable Misalignment	Using costs/visits to approximate health needs	Systemic underestimation of actual disease burden	Low-income and uninsured groups	31,649,194

PHM, proactive health management; SDoH, social determinants of health; PGHD, patient-generated health data; SES, socioeconomic status; PPG, photoplethysmography; ERM, empirical risk minimization; PMID, PubMed identifier.

## 4 The impact of algorithmic bias on health equity: from individual differences to systemic inequities

Prediction bias manifests as health outcome inequities in real-world deployment. The consequences extend far beyond statistical decline—they alter the distribution of clinical resources and undermine the social trust on which long-term monitoring depends (56, 57). PHM structurally links continuous data collection with threshold-based clinical intervention and applies standard algorithms to inherently unequal social data, thereby generating systematically unequal clinical outcomes. Therefore, the impact of algorithmic bias in PHM should not be understood merely as reduced predictive accuracy, but as a mechanism through which unequal data representation is translated into unequal prevention opportunities, unequal resource allocation, and weakened institutional legitimacy. This section analyzes these consequences at three levels: differential health outcomes, mismatch in preventive resource allocation, and the self-reinforcing feedback loop between trust erosion and data poverty.

### 4.1 Differences in health outcomes: the imbalance between risk identification and prevention opportunities

The core goal of PHM is to identify high-risk individuals before adverse clinical events occur. The high FNR among vulnerable groups hinders equitable early intervention opportunities (46, 58). Such misclassification reflects a scarcity of representation within training data rather than an absence of genuine biological risk. Patients at underestimated risk miss the opportunity to strengthen clinical follow-up, lifestyle guidance or automatic early warning. In this sense, predictive bias does not only generate an incorrect numerical score; it determines whether preventive care is received at all, and shapes its timing and intensity when it is.

The consequences of subgroup miscalibration can be illustrated through cardiovascular risk prediction, one of the most widely studied domains in clinical risk stratification. External validation studies of cardiovascular risk prediction tools have demonstrated that model performance can vary substantially across population subgroups. An independent external validation of QRISK3 using UK Biobank data found that the model systematically overpredicted cardiovascular disease risk, particularly in older participants, by as much as 20%, indicating that risk tools may not transport evenly across populations with different demographic and health profiles (59). Similarly, external validation of SCORE2 in an ethnically and socioeconomically diverse population in the Netherlands found that model performance varied across subgroups, reinforcing the need to account for demographic and social heterogeneity when risk scores are applied in clinical decision-making (60). Whether miscalibration takes the form of systematic underestimation or overestimation, the consequence for clinical decision-making is the same: patients whose predicted risk diverges from their actual risk burden may not receive the preventive care their clinical situation warrants. This matters directly because cardiovascular risk scores are used to determine whether patients reach thresholds for statin therapy, blood pressure management, lifestyle counselling, intensive follow-up and specialist referral—meaning that subgroup miscalibration translates directly into foregone or misdirected preventive opportunities.

The potential harm is therefore not limited to statistical misclassification. If the risk of a woman, an older patient, an ethnic minority patient or a socioeconomically disadvantaged patient is underestimated, that patient may remain below the intervention threshold despite carrying a clinically meaningful risk burden. For PHM, this problem is particularly serious because the value of the system depends on acting before deterioration occurs. Once a high-risk patient is incorrectly classified as low-risk, the opportunity for early prevention may be lost before the health system recognizes the severity of the condition.

The clinical path triggered by the threshold makes this problem more prominent. When a patient’s predicted risk approaches the intervention threshold, even small prediction errors determine clinical pathways—whether to receive intensive monitoring or standard care (55, 61). The deviation in this link determines the specific clinical path, not the abstract statistical difference. In high-risk applications such as automatic clinical early warning or intensive care upgrade, the clinical impact of predictive bias and resulting inequities is more pronounced than basic behavior tracking applications. This mechanism reveals a temporal dimension of health inequity. Some patients receive preventive intervention before clinical deterioration, whereas others are recognized only after avoidable progression, emergency admission or acute complications. Algorithmic bias therefore affects not only who is identified as high-risk, but also when they become visible to the health system.

### 4.2 Resource mismatch: the concentration and cumulative advantages of preventive services

Prediction bias shapes the results of risk stratification at the population level, which in turn affects the way preventive resources are allocated in the health system. Patients with complete and continuous electronic medical records are more likely to be identified and included in disease management projects; high-risk groups with sparse or fragmented data are therefore omitted (62, 63). Differential data representation translates into differential access to preventive healthcare services. Preventive services are concentrated in groups that already have the ability to manage health.

This produces a cumulative advantage effect. Individuals who are already digitally connected, regularly followed up, insured, and well documented become more visible to PHM systems, while individuals with unstable access to care, fragmented records, language barriers, lower digital literacy or limited device access remain algorithmically less visible. As a result, preventive care may become concentrated among populations that are already better positioned to benefit from it.

There is a “health volunteer effect” in large databases such as the UK Biobank—cohort participants subjected to digital monitoring tend to have better health status, higher compliance, and greater economic resources than the actual target population (64), meaning PHM models trained on such data may systematically underserve those most in need. A related distortion arises through proxy variable choice: economic barriers reduce healthcare utilization among low-income populations, thereby artificially depressing their predicted risk scores (65). The research of Obermeyer et al. demonstrated that this proxy mechanism systematically redirects care management resources toward patients with better actual health status, rather than those with greater clinical need (52).

Resource mismatch therefore operates through both data visibility and target definition. Patients who interact frequently with healthcare systems generate more data and are more likely to be selected for preventive programs, while patients who face structural barriers to care may appear less risky precisely because their needs are less frequently documented. Resource mismatch eventually leads to an increase in preventable hospitalization and emergency visits—outcomes that PHM systems are designed to prevent. When preventive resources are guided by biased risk scores, PHM may paradoxically reinforce downstream acute-care demand: the system may appear efficient at the level of algorithmic allocation, while becoming inequitable at the level of population health outcomes.

### 4.3 Self-reinforcement of trust erosion and data poverty

The continuous operation of the PHM system depends on the long-term active participation of users. Patients need to continue to interact with wearable devices and algorithm platforms for several years. Trust in relevant institutions declines when the risk assessment obtained by marginalized groups does not match their actual health status or life situation (66, 67). After repeatedly experiencing unreliable evaluation results, this distrust has evolved into a general rejection of AI tools. Patient disengagement manifests as reduced device compliance, decreased symptom reporting, and withdrawal from long-term monitoring programs.

Research on medical AI acceptance and digital health adoption supports this dynamic. Recent studies have identified trust as a central condition for the acceptance of AI-enabled healthcare systems, particularly when patients and clinicians are asked to rely on opaque or partially explainable algorithmic recommendations (68, 69). For historically marginalized groups, this issue is especially salient: prior experiences of unequal treatment or exclusion from healthcare may heighten sensitivity to inaccurate or poorly explained algorithmic outputs, making trust more fragile and harder to rebuild once lost.

Beyond institutional trust, the specific design properties of AI systems also shape whether users regard algorithmic outputs as legitimate. Explainability, fairness, privacy protection, robustness and accountability have been identified as central requirements for trustworthy medical AI (70, 71). When these properties are absent or perceived to be absent, patients may question whether the system serves their interests—and that questioning can translate directly into disengagement.

In PHM, trust is therefore not only an attitude toward technology; it is a prerequisite for data generation. Patients are expected to wear devices, report symptoms, respond to alerts and consent to the reuse of longitudinal health data. If patients believe that the system does not represent them or produces recommendations that conflict with their lived experience, their willingness to participate may decline. Each of these forms of disengagement reduces the quality and completeness of data contributed by marginalized groups to the PHM system (47).

Behavioral loss directly weakens the data infrastructure. The decrease in device compliance and the increase in withdrawal rate limit the inclusion of effective data of minority groups (72–74). Model performance declines further, prompting more patients to disengage. It forms a self-reinforcing feedback loop: unequal data representation leads to biased prediction; biased prediction produces unreliable or unfair user experiences; unreliable experiences erode trust and reduce participation; reduced participation deepens data poverty; and data poverty further entrenches the underrepresentation that initiated the cycle.

Trust erosion is therefore not merely an ethical or communication problem—it is a data-generating mechanism that reshapes the future training environment of PHM systems. As marginalized populations disengage from digital monitoring, their risks become progressively less visible to future models, and the system becomes increasingly calibrated toward those who remain continuously connected. The equity challenge in PHM thus stems from long-term cumulative structural inequalities rather than isolated process errors, which makes application results substantially deviate from the core goal of public health inclusion.

This feedback loop also affects institutional legitimacy. PHM systems are often justified by their promise to provide earlier, more personalized, and more inclusive prevention. However, if marginalized groups repeatedly experience inaccurate assessment, missed intervention, or exclusion from preventive services, the legitimacy of both algorithmic systems and the health institutions deploying them will be weakened. Table 2 summarizes how these three consequences—differential health outcomes, resource mismatch, and trust erosion—translate into downstream impacts on patients, health systems, and institutional legitimacy, illustrating how algorithmic bias in PHM operates not as a technical malfunction but as a structural mechanism of health inequity.

**Table 2:** Inequitable consequences of predictive bias and their public health implications.

Dimension	Manifestation	Underlying mechanism	Public health implication
Health outcomes	Missed or delayed identification of high-risk individuals in vulnerable subgroups	Disparities in recall and elevated false-negative rates lead to missed early intervention windows	Increased avoidable morbidity and delayed preventive care
Resource allocation	Reinforcement of cumulative advantage in health care	Output-driven allocation based on biased risk predictions preferentially benefits data-rich populations	Preventive resources are diverted away from high-risk but underrepresented groups
Trust	Algorithm aversion, device disengagement, and declining user adherence	Recurrent misclassification and opaque decision-making undermine trust in PHM systems	Reduced participation and the formation of self-reinforcing data poverty feedback loops

PHM, proactive health management.

## 5 Systematic response: equity intervention at the level of data, algorithms and governance

Isolated algorithm adjustment cannot solve the systemic equity challenge in PHM. Prediction bias originates in the data generation stage and continues to accumulate during model development, clinical deployment and post-deployment monitoring. Isolated interventions at single points are usually ineffective (75, 76). Effective response requires the establishment of a governance framework covering the entire life cycle of technology: addressing representativeness deficits at the data collection stage, constraining bias amplification during model development, and establishing clear accountability mechanisms in clinical applications. However, these interventions cannot be understood as purely technical corrections. Their effectiveness depends on whether healthcare systems have the practical capacity to implement them, including interoperable data infrastructure, workflow integration, continuous monitoring capacity, institutional accountability and patient trust (77, 78). Compared with existing lifecycle-oriented AI governance approaches that primarily emphasize responsible model development, evaluation, and oversight, the present framework extends this perspective by operationalizing the self-reinforcing feedback between data poverty, prediction bias, and trust erosion identified in Section 4 as a central governance target. By integrating data-level, algorithm-level, and governance-level interventions into a closed-loop system, this framework transforms fairness from a discrete evaluation requirement into a continuous, adaptive process throughout the PHM lifecycle.

**Figure 2:**
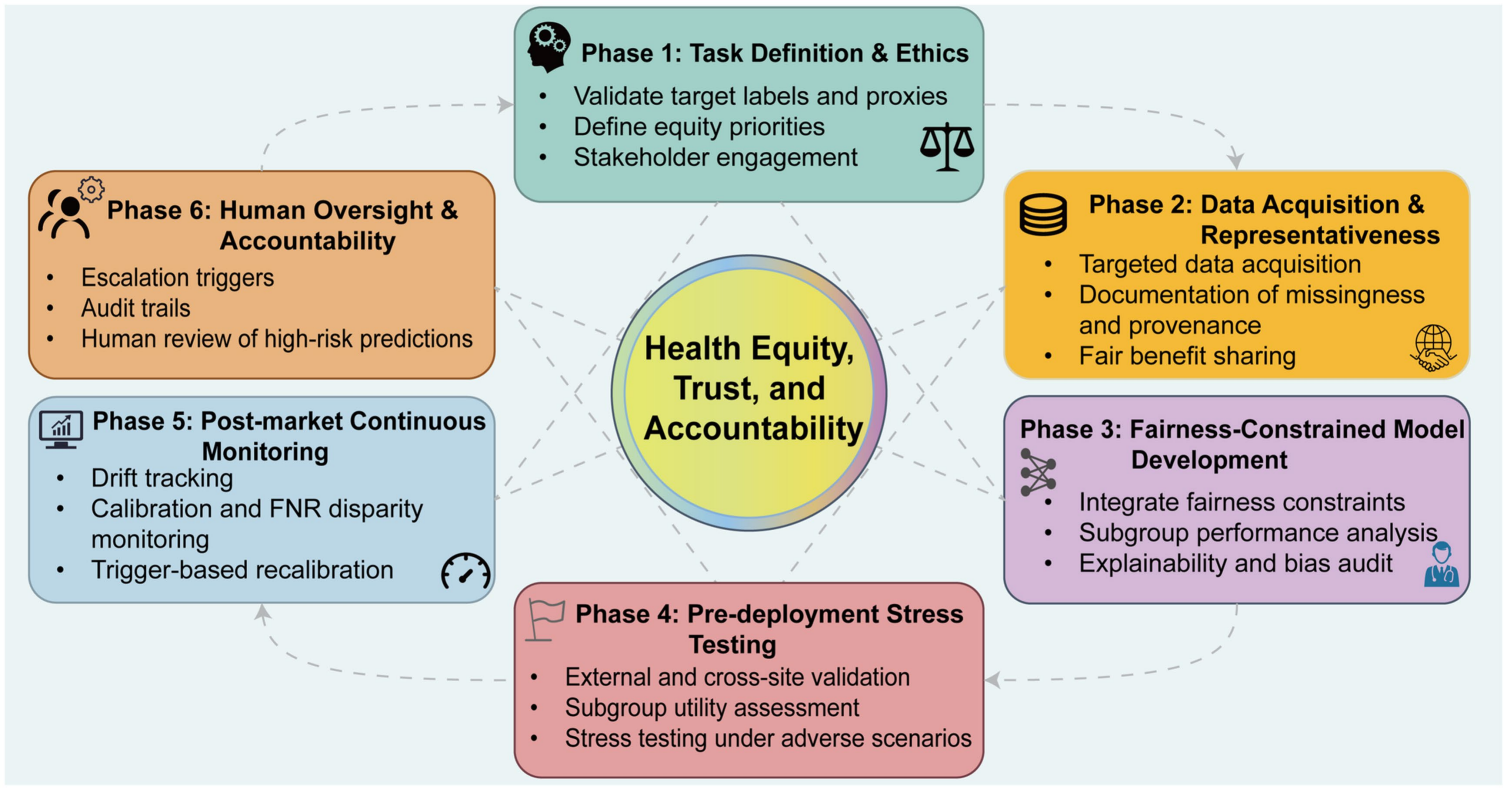
Closed-loop governance framework for equity in proactive health management. This figure presents a six-phase closed-loop governance framework for advancing health equity throughout the proactive health management (PHM) lifecycle. The framework integrates data governance, algorithmic safeguards, and continuous monitoring to address algorithmic bias at its source. The first three phases establish the foundation: Phase 1 (Task Definition & Ethics) validates target labels and fairness priorities through stakeholder engagement; Phase 2 (Data Acquisition &Representativeness) implements targeted data collection and documents data provenance; and Phase 3 (Fairness-Aware Model Development) embeds fairness constraints and conducts subgroup audits. Phases 4–6 operationalize accountability: Phase 4 (Pre-deployment Stress Testing) conducts external validation and assesses performance across subgroups; Phase 5 (Post-market Continuous Monitoring) tracks model drift and triggers recalibration when warranted; and Phase 6 (Human Oversight & Accountability) maintains escalation protocols and human review of high-risk predictions. The cyclical design reflects the iterative nature of equity governance, with continuous feedback loops enabling adaptive refinement of data strategies, model parameters, and deployment protocols. PHM, proactive health management; FNR, false negative rate.

The setting of equity goals should adopt a risk-based approach and differentiate the configuration according to the characteristics of specific clinical tasks. For high-risk systems such as automated early warning or triage, the development strategy should give priority to ensuring the diagnostic safety of vulnerable groups and reducing the risk of missed diagnosis. Low-risk behavioral intervention tools allow trade-offs between predictive accuracy and fairness constraints (56, 62). Directly aligning equity goals with task characteristics can help build consensus among data scientists, clinicians and regulatory agencies, and can improve interdisciplinary collaboration during model development and deployment. Figure 2 summarizes the core stage of this life cycle framework, and the following sections are expanded separately.

### 5.1 Data level: from passive accumulation to target representation

Predictive bias is primarily driven by non-random missingness in PGHD, and it is difficult to solve this problem by simply increasing the sample size. Incomplete monitoring and interruption of follow-up are common in the health records of marginalized groups. Indiscriminate data expansion mainly increases the sampling frequency of the dominant population, and this unbalanced growth widens the data gap for underrepresented subgroups (79, 80). Advancing health equity requires the data collection mode to shift from passive accumulation to purposeful sampling, and to accurately allocate limited quality control resources to underrepresented subgroups (81). In other words, the key issue is not only how much data PHM systems collect, but whose data are repeatedly collected, whose data are missing, and whether missingness itself reflects unequal access to digital monitoring and healthcare services (82).

A targeted representation strategy does not conflict with the random sampling principle. On the basis of ensuring the representativeness of the baseline population, a dedicated validation cohort is introduced for specific subgroups that are prone to calibration drift to strengthen the robustness of the model (83, 84). It is necessary to be cautious when dealing with missing data related to SDoH, and routine statistical imputation is difficult to bridge the deep gap. This missing pattern reflects structural inequalities such as digital access barriers or specific employment systems, which go beyond the scope of random technical failures (83, 84). If these characteristics are regarded only as routine missing items, social disadvantages may be solidified in algorithmic logic, inducing automated bias and exacerbating existing health disparities (47, 82).

Active learning can identify subgroups with high predictive uncertainty, and synthetic data augmentation strengthens the model’s ability to characterize diverse populations by incorporating rare clinical patterns (85, 86). However, synthetic data should be used cautiously. If generative models are trained on biased source data, synthetic samples may reproduce or amplify the same representational imbalance rather than correct it. Therefore, synthetic data augmentation should be accompanied by subgroup validation, external testing and transparent documentation of data provenance (18). Longitudinal cardiovascular risk monitoring shows that the data gaps and monitoring interruptions of older adults and low-income patients are significant. Identifying these specific missing mechanisms in advance is more effective than post-hoc statistical correction. The distribution audit before deployment identifies variable offsets that may induce threshold failure, replacing manual statistical balancing methods (87, 88). This practice ensures that the training set accurately reflects the structural risk characteristics of vulnerable groups.

A useful illustration of why data quantity alone is insufficient comes from the “healthy volunteer” problem documented in large biomedical cohort studies. Although large datasets may appear statistically powerful, they may still underrepresent individuals with lower socioeconomic status, poorer baseline health, limited digital access or lower willingness to participate in long-term monitoring (64). For PHM, this means that expanding dataset size alone cannot guarantee equity if the additional data mainly come from already advantaged and highly engaged participants. Data-level intervention should therefore include subgroup-specific recruitment, device support, community engagement and dataset documentation before model development begins.

### 5.2 Algorithm level: clinical utility and constraint optimization of subgroups

Only looking at the excellent overall performance index hides the deterioration of performance at the subgroup level. Under non-independent and identically distributed (Non-IID) conditions, ERM training optimizes performance for the majority class while suppressing minority-specific risk signals due to their sparse representation (89, 90). The evaluation framework goes beyond a single overall indicator and focuses on analyzing the clinical utility across specific demographic subgroups. Stratified sensitivity, the difference in false negative rate and the net benefit within the decision-making threshold are directly related to the safety evaluation of vulnerable patients in automated intervention (91). Therefore, model evaluation should report not only aggregate discrimination, but also subgroup calibration, false negative rate differences, threshold-dependent net benefit and clinical consequences of errors (92).

Fairness constraints can be directly embedded in the training stage. Regularization techniques or equal opportunity constraints can reduce subgroup differences in false-negative rates and suppress the deviation driven by the majority group (93, 94). Such interventions may introduce performance trade-offs, manifesting as reduced overall accuracy or uneven improvement in minority performance. These trade-offs should be explicitly documented and clinically justified to ensure that fairness constraints align with medical reasoning rather than purely statistical optimization. Real-world evaluation and threshold-specific sensitivity analyses are therefore needed to determine whether fairness-oriented model improvements translate into clinically meaningful benefits across subgroups (95). This is particularly important because fairness metrics may conflict with one another in clinical prediction. For example, calibration, equal opportunity and predictive parity may not be simultaneously achievable when baseline risks differ across population groups. The choice of fairness constraint should therefore be clinically justified rather than treated as a purely mathematical decision (92, 96).

The algorithm does not blindly eliminate all group differences. Some of the observed differences reflect the real biological heterogeneity or environmental exposure, and the cause is independent of structural discrimination (97). The removal of group information that lacks clinical basis damages the signals with diagnostic value. The development process integrates explainable tools such as Shapley Additive Explanations (SHAP) to help epidemiologists distinguish between structural deviations that need to be mitigated and reasonable clinical heterogeneity that requires subgroup calibration (98, 99). However, explainability alone is not sufficient. A model may be interpretable but still inequitable if its target variable, proxy variables or deployment thresholds encode structural disadvantage. Therefore, explainable analysis should be combined with target-variable review, subgroup validation and clinical review of whether the model’s predictions correspond to actual health need (47).

The population health management algorithm analyzed by Obermeyer et al. illustrates this point directly. The algorithm used healthcare cost as a proxy for health need, which systematically underestimated the illness burden of Black patients because historical healthcare spending reflected unequal access to care rather than true clinical need. This case shows that algorithmic bias may arise not only from model architecture, but also from the definition of the prediction target itself (52). For PHM systems, predicting cost, utilization or engagement may be administratively convenient, but such targets require explicit equity review and subgroup validation before they are used to trigger preventive intervention or resource allocation.

External validation is equally important before deployment. A widely implemented proprietary sepsis prediction model was externally validated by Wong et al., who found that its real-world performance was substantially lower than expected, raising concerns about using such systems without local validation and continuous monitoring (45). This case is directly relevant to PHM because predictive tools are often transferred across institutions with different populations, documentation practices, device-use patterns and clinical workflows. A model that appears acceptable in the development environment may become inaccurate or inequitable after deployment. Therefore, subgroup-specific external validation and post-deployment recalibration should be treated as core requirements rather than optional technical refinements (45, 77).

### 5.3 Governance level: from ethical advocacy to life cycle supervision

Health equity in digital healthcare requires continuous governance mechanisms that adapt to changing population structures, technology compliance, and policy contexts. Population structure changes, equipment compliance fluctuations and policy adjustments continuously affect the clinical efficacy of the prediction model (100). Institutional governance goes beyond one-time ethical certification and establishes a self-correction mechanism with continuous monitoring and audit capabilities. This life-cycle orientation is consistent with recent reporting and evaluation frameworks for clinical AI, which emphasize transparent reporting, prospective evaluation, human factors and post-deployment monitoring rather than one-time model approval (101, 102).

Pre-deployment evaluation is not limited to a single accuracy indicator. Regulatory review conducts subgroup analysis, focusing on calibration and clinical practicality under specific decision-making thresholds (103). The selection of proxy variables must be included in regulatory scope. Proxies such as historical medical costs—which correlate with socioeconomic factors rather than actual health needs—systematically encode structural inequalities into triage algorithms (104). Proxy variable review should be integrated into pre-deployment evaluation to align with emerging global standards, including the European Union (EU) Artificial Intelligence Act and the Food and Drug Administration (FDA) medical software lifecycle management framework (105, 106). In practice, this means that institutions should evaluate not only whether a model predicts well, but also what it predicts, for whom it predicts reliably, and whether the prediction target is ethically and clinically appropriate (52).

After deployment, health equity indicators—including fairness metrics at subgroup levels and health outcome disparities—should be integrated into continuous post-market surveillance. When there is a calibration shift or missed diagnosis rate in a specific population, structured clinical review is automatically triggered (107). High-risk triage scenarios still need to maintain HITL oversight; however, since clinicians themselves have cognitive biases, systematic documentation and audit trails are essential to constrain deviations in human judgment (19). Human oversight should therefore not be treated as a simple safeguard that automatically corrects machine bias. Clinicians may also be affected by alert fatigue, workload pressure, prior assumptions about patients and unequal access to contextual information. For this reason, human-in-the-loop governance requires structured override documentation, regular audit of clinician responses and clear escalation pathways (102).

The key to sustaining long-term participation in PHM cohort studies is ensuring that individuals who contribute data can meaningfully benefit from doing so. An equitable benefit-sharing mechanism should therefore be designed to encourage the sustained participation of vulnerable groups (108). This is especially important for PHM because long-term data contribution depends on sustained trust. If marginalized groups provide data but do not receive timely feedback, meaningful preventive services or transparent explanations of algorithmic recommendations, participation may decline and the data-poverty cycle described in Section 4.3 may continue. Achieving this requires transforming abstract equity principles into specific, auditable and enforceable workflows that address algorithmic bias and prevent the self-reinforcing cycle of data scarcity and health inequities.

### 5.4 Implementation barriers

Although the data, algorithm and governance interventions described above provide a structured response to algorithmic bias in PHM, their implementation in real-world health systems faces significant practical constraints. Equity-oriented PHM requires methodological improvement as a foundation, but successful implementation also depends on sufficient institutional capacity, interoperable data infrastructure, sustainable funding and clearly assigned accountability—conditions that are often unmet in resource-constrained settings (77, 78). Without these enabling conditions, even well-designed technical interventions may fail to produce equitable outcomes in practice.

At the data level, collecting more representative PGHD requires active outreach to underrepresented populations, yet these groups often face compounding barriers including limited digital access, privacy concerns, language differences and lower institutional trust. Variables that are essential for equity auditing—such as race, ethnicity, income or housing instability—may be incomplete, legally sensitive or inconsistently recorded across institutions, limiting the feasibility of subgroup monitoring even when the intention exists (22). Privacy protection requirements may further create tension with equity auditing, since fairness assessment often depends on collecting and analyzing socially sensitive characteristics. Resolving this tension does not require abandoning equity auditing, but rather designing governance frameworks that protect privacy while enabling fairness assessment. Practical governance should therefore combine data minimization with transparent consent procedures and independent oversight of sensitive-variable use (47).

At the governance level, accountability fragmentation remains a central barrier. PHM systems are typically developed by technology vendors, procured by healthcare institutions, used by clinicians and regulated by external agencies. When biased predictions lead to missed prevention or unequal resource allocation, responsibility may become dispersed across multiple actors. Effective governance should therefore specify accountability across the model lifecycle—from data collection and model development through deployment, monitoring and patient communication—and embed equity monitoring into existing quality-improvement systems with clearly defined escalation procedures (77, 78).

## 6 Conclusion

### 6.1 Main findings

The equity challenge in PHM is not an isolated technical defect but a lifecycle problem spanning data generation, model development, clinical deployment and governance. This review identifies three recurring mechanisms: non-random representativeness deficits in PGHD and clinical records, amplification of upstream data gaps into subgroup performance disparities during model development, and value misalignment in proxy variable selection and prediction target definition. Together, these mechanisms create a self-reinforcing cycle in which disadvantaged populations remain underrepresented in data, receive less accurate predictions and experience fewer preventive interventions, further deepening existing inequities. Breaking this cycle requires coordinated intervention across data, algorithm and governance levels rather than isolated algorithmic correction.

### 6.2 Limitations

As a narrative review, this study may be subject to selection bias and does not claim exhaustive coverage. The search was limited to three databases and to English-language publications, which may underrepresent evidence from non-Anglophone health systems. The heterogeneity of included studies in terms of clinical settings, populations, and fairness definitions limits direct comparison, and the absence of quantitative synthesis means that the relative effectiveness of specific interventions cannot be formally estimated. Much of the available evidence is drawn from high-income settings, and transferability to resource-constrained contexts requires further validation. No formal risk-of-bias appraisal was applied to individual included studies beyond the overall methodological guidance provided by the SANRA criteria, which may limit the interpretability of evidence quality across the review.

### 6.3 Future directions

Future research should address the specific gaps identified in this review. Building on the representativeness deficits discussed in Sections 3.1 and 5.1, studies should develop better methods to characterize structural missingness in PGHD and SDoH variables, and evaluate whether synthetic data generation reduces or amplifies existing bias. Notably, the process of extracting latent SDoH indicators—such as housing instability and caregiving burden—from clinical text records carries an inherent risk of introducing new measurement error; this risk should therefore be explicitly incorporated into study design protocols rather than treated as a post-hoc concern. The privacy implications of integrating sensitive social variables into automated triage workflows also warrant dedicated methodological attention.

Building on the subgroup performance problems identified in Sections 3.2 and 5.2, future work should test subgroup-level calibration, threshold-specific clinical utility, and clinically justified fairness trade-offs. Health equity assessment should extend beyond static pre-deployment fairness benchmarking to encompass continuous post-deployment audit mechanisms. Such mechanisms should be capable of tracking subgroup coverage gaps, monitoring calibration stability over time, and evaluating clinical utility across decision-making thresholds. Crucially, monitoring protocols should be linked to pre-specified action triggers that define when model recalibration is warranted, when the clinical scope of application should be restricted, and when automated decision support should be suspended—ensuring that oversight in large-scale population health interventions is governed by systematic assessment criteria rather than *ad hoc* review.

Building on the value misalignment and governance gaps in Sections 3.3, 5.3, and 5.4, empirical studies should examine proxy-variable review processes, accountability assignment structures, and privacy-compatible equity auditing in real-world PHM workflows. At the institutional level, hospitals and developers should establish traceable documentation systems recording prediction target definitions, proxy variable selection rationale, and threshold-setting decisions. Regulatory bodies should likewise move beyond voluntary commitments by designating post-deployment monitoring as a mandatory requirement within normative standards. Two empirical questions are particularly pressing and remain underexplored: first, how algorithmic bias propagates into and distorts clinician judgment in hybrid decision-making environments; and second, whether algorithmically assisted clinical decisions produce systematically differential outcomes across population subgroups. Both questions should be prioritized on the agenda of future empirical research.

### 6.4 Achieving health equity in proactive health management

This review contributes a lifecycle perspective integrating data representativeness, algorithmic fairness, and governance accountability into a coherent analytical framework. The value of PHM should be assessed not only by predictive accuracy but also by whether it reduces preventable disparities and maintains trust across populations. By transitioning fairness from a post-hoc technical correction to an ex-ante operational priority, the proposed closed-loop governance framework provides an actionable pathway to disrupt self-reinforcing data poverty feedback loops. When equity governance is operationalized as a continuous, multi-phase systemic capability, PHM technologies can genuinely fulfill their public health promise and advance clinical outcomes for the global populations who need them most.

## Glossary

AI: Artificial Intelligence
AUROC: Area Under the Receiver Operating Characteristic Curve
EHR: Electronic Health Record
eGFR: Estimated Glomerular Filtration Rate
ERM: Empirical Risk Minimization
EU: European Union
FDA: Food and Drug Administration
FNR: False-Negative Rate
HITL: Human-in-the-Loop
ML: Machine Learning
MNAR: Missing Not at Random
NHS: National Health Service
Non-IID: Non-Independent and Identically Distributed
PGHD: Patient-Generated Health Data
PHM: Proactive Health Management
PPG: Photoplethysmography
SANRA: Scale for the Assessment of Narrative Review Articles
SDoH: Social Determinants of Health
SES: Socioeconomic Status
SHAP: Shapley Additive Explanations
