# Supplementary material for: Advancing health equity in proactive health management: from data underrepresentation and algorithmic bias to a closed-loop governance framework
Source: Front Public Health. 2026 Jun 17;14:1863050. doi: 10.3389/fpubh.2026.1863050 (PMC13318946; doi:10.3389/fpubh.2026.1863050)
Supplement: Supplementary file 1 [file Data_Sheet_1.DOCX]

Supplementary Material

# Supplementary Material S1: Platform-Specific Database Search Strings

**PubMed**

(“proactive health management” OR “wearable device*” OR “patient-generated health data” OR “PGHD” OR “mobile health” OR “mHealth” OR “remote patient monitoring”) AND (“artificial intelligence” OR “machine learning” OR “deep learning” OR “predictive model*” OR “algorithmic*” OR “clinical decision support”) AND (“algorithmic bias” OR “algorithmic fairness” OR “health equity” OR “health disparit*” OR “digital divide” OR “social determinants of health” OR “underrepresentation”)

MeSH terms additionally applied: Health Equity [MeSH], Artificial Intelligence [MeSH], Wearable Electronic Devices [MeSH], Healthcare Disparities [MeSH], Social Determinants of Health [MeSH]

Date range: January 1, 2015 – February 28, 2026

**Web of Science**

TS=(“proactive health management” OR “wearable device*” OR “patient-generated health data” OR “PGHD” OR “mobile health” OR “mHealth” OR “remote patient monitoring”) AND TS=(“artificial intelligence” OR “machine learning” OR “deep learning” OR “predictive model*” OR “algorithmic*” OR “clinical decision support”) AND TS=(“algorithmic bias” OR “algorithmic fairness” OR “health equity” OR “health disparit*” OR “digital divide” OR “social determinants of health” OR “underrepresentation”)

Date range: January 1, 2015 – February 28, 2026

**Scopus**

TITLE-ABS-KEY(“proactive health management” OR “wearable device*” OR “patient-generated health data” OR “PGHD” OR “mobile health” OR “mHealth” OR “remote patient monitoring”) AND TITLE-ABS-KEY(“artificial intelligence” OR “machine learning” OR “deep learning” OR “predictive model*” OR “algorithmic*” OR “clinical decision support”) AND TITLE-ABS-KEY(“algorithmic bias” OR “algorithmic fairness” OR “health equity” OR “health disparit*” OR “digital divide” OR “social determinants of health” OR “underrepresentation”)

Date range: January 1, 2015 – February 28, 2026
